# Meniscectomy is associated with a higher rate of osteoarthritis compared to meniscal repair following acute tears: a meta-analysis

**DOI:** 10.1007/s00167-023-07600-y

**Published:** 2023-10-09

**Authors:** Filippo Migliorini, Luise Schäfer, Andreas Bell, Christian David Weber, Gianluca Vecchio, Nicola Maffulli

**Affiliations:** 1 Department of Orthopaedic, Trauma, and Reconstructive Surgery, RWTH University Hospital, Pauwelsstraße 30, 52074 Aachen, Germany; 2 Department of Orthopedics and Trauma Surgery, Academic Hospital of Bolzano (SABES-ASDAA), Teaching Hospital of Paracelsus Medical University, 39100 Bolzano, Italy; 3 Department of Orthopaedic and Trauma Surgery, Eifelklinik St.Brigida, 52152 Simmerath, Germany; 4 Department of Medicine, Surgery and Dentistry, University of Salerno, 84081 Baronissi, Italy; 5 Faculty of Medicine and Psychology, University Hospital Sant’ Andrea, University La Sapienza, 00185 Rome, Italy; 6 School of Pharmacy and Bioengineering, Faculty of Medicine, Keele University, Stoke On Trent, ST4 7QB UK; 7 Barts and the London School of Medicine and Dentistry, Centre for Sports and Exercise Medicine, Queen Mary University of London, Mile End Hospital, London, E1 4DG UK

**Keywords:** Knee, Meniscus, Repair, Resection, Meniscectomy, Osteoarthritis

## Abstract

**Purpose:**

Meniscal tears are common and may impair knee function and biomechanics. This meta-analysis compared meniscal repair versus resection in patients with symptomatic meniscal tears in terms of patient-reported outcomes measures (PROMs), joint width, surgical failure, and rate of progression to osteoarthritis (OA) at conventional radiography.

**Methods:**

This study was conducted according to the 2020 PRISMA statement. In August 2023, the following databases were accessed: PubMed, Web of Science, Google Scholar, and Embase. Two reviewers independently performed the analysis and a methodological quality assessment of the included studies. All the clinical investigations which compared repair versus resection of meniscal tears were accessed.

**Results:**

Data from 20 studies (31,783 patients) were collected. The mean BMI was 28.28 ± 3.2 kg/m^2^, and the mean age was 37.6 ± 14.0 years. The mean time elapsed from injury to surgery was 12.1 ± 10.2 months and the mean medial joint width was 4.9 ± 0.8 mm. Between studies comparability at baseline was found in age, women, BMI, time from injury to surgery and length of the follow-up, PROMs, medial joint width, and stage of OA. The resection group demonstrated a greater Lysholm score (*P* = 0.02). No difference was found in the International Knee Documentation Committee (*P* = 0.2). Nine studies reported data on the rate of failures at a mean of 63.00 ± 24.7 months. No difference was found between the two groups in terms of persistent meniscal symptoms (*P* = 0.8). Six studies reported data on the rate of progression to total knee arthroplasty at a mean of 48.0 ± 14.7 months follow-up. The repair group evidenced a lower rate of progression to knee arthroplasty (*P* = 0.0001). Six studies reported data on the rate of advanced knee OA at a mean of 48.0 ± 14.7 months of follow-up. The repair group evidenced a lower rate of advanced knee OA (*P* = 0.0001). No difference was found in the mean joint space width (*P* = 0.09).

**Conclusion:**

Meniscal repair is associated with a lower progression to knee osteoarthritis at approximately six years of follow-up compared to partial meniscectomy. No difference in PROMs, medial joint width, and failures were evidenced.

**Level of evidence:**

Level III, meta-analysis.

## Introduction

Meniscal tears are common and increase with age [41, 57]. Squatting, kneeling, crawling, chair sitting while driving, stair climbing, lifting items, and walking are associated with acute meniscal tears [39, 61, 79]. Both the lateral and medial menisci help preserve the knee biomechanics, and are important for shock absorption, joint stability, joint lubrication, and proprioception [6, 66, 90]. Meniscal tears have different aetiologies and injury patterns [44, 47, 60]. First, a distinction is made between acute and degenerative tears [39, 56]. Acute tears are commonly the result of trauma or sports injuries. The development of degenerative tears is caused by increasing age, chronic joint instability, and malalignment [15, 52, 58, 96]. When planning management, the shape, form, and location of the tears must be considered [14, 57]. The most common tear patterns are horizontal tears, bucket-handle tears, longitudinal tears, oblique or flap tears, radial tears, meniscal root tears, and complex tears consisting of a combination of different tear morphologies [3, 10, 13, 67]. Total or partial meniscectomy was considered the gold standard in the management of meniscal injuries [9, 21]. However, the loss of meniscal function and the altered biomechanics of the knee have caused a concern [32, 53]. In this context, the number of clinical studies evaluating strategies for meniscal repair has recently increased [1, 11, 22, 54]. In the past few decades, several techniques for meniscal repair have been advocated [28, 80, 89]. Meniscal repair led to a satisfactory healing rate, restoring knee biomechanics and function, and preventing the development of long-term complications [9, 87]. However, arthroscopic meniscectomy is still commonly performed, and the clinical advantages of meniscal repair are often undervalued.

This meta-analysis compared meniscal repair versus resection in patients with symptomatic meniscal tears in terms of patient-reported outcomes measures (PROMs), joint width, surgical failure, and rate of progression to osteoarthritis (OA) at conventional radiography. It was hypothesised that meniscal repair performs better compared to meniscal resection.

## Methods

### Eligibility criteria

All the clinical investigations which compared repair versus resection of meniscal tears were accessed. Only studies published in peer reviewed journals were considered. According to the authors’ language capabilities, articles in English, German, Italian, French and Spanish were eligible. Only studies with level I to IV of evidence, according to Oxford Centre of Evidence-Based Medicine [37], were considered. Studies which enhanced meniscal surgery with regenerative therapies (e.g., platelet rich plasma, mesenchymal stem cells) were not included. All types of repairs were included irrespective of the surgical technique and materials. All types of meniscal tears were considered, irrespective to their aetiology, previous conservative management, location, or extend. Only studies with a minimum of 12 months of follow-up were considered. Reviews, opinions, letters, and editorials were not considered. Animals, in vitro, biomechanics, computational, and cadaveric studies were not eligible, nor were those evaluating experimental physiotherapeutic protocols. Missing quantitative data under the outcomes of interests warranted the exclusion of the study.

### Search strategy

This study was conducted according to the Preferred Reporting Items for Systematic Reviews and Meta-Analyses: the 2020 PRISMA statement [69]. The following PICOT algorithm was established:

- P (Problem): meniscal tears;
- I (Intervention): meniscal repair;
- C (Comparison): meniscal resection;
- O (Outcomes): PROMs, medial joint width, rate of OA and failure;
- T (Timing): minimum 12 months of follow-up.

In August 2023, the following databases were accessed: PubMed, Web of Science, Google Scholar, and Embase with no time constrain. The algorithm used for the literature search is shown in supplementary material.

### Selection and data collection

Two authors (**;**) independently performed the database search. All the resulting titles were screened by hand and the abstracts were accessed. The full text of the abstracts which matched the topic of interest was accessed. A cross reference of the bibliography of the full-text articles was also performed for inclusion. Disagreements were debated and mutually solved by the authors. In case of further disagreements, a third senior author (**) took the final decision.

### Data items

Two authors (**;**) independently performed data extraction. The following data at baseline were extracted: author, year of publication and journal, length of the follow-up, number of patients and their mean age and BMI. Data concerning the following PROMs were collected at baseline and at last follow-up: Lysholm Knee Scoring Scale [51] and International Knee Documentation Committee (IKDC) [35]. The minimum clinically important difference (MCID) for the Lysholm score was 10/100 and 15/100 for the IKDC [2, 40, 64]. Data on the width medial joint compartment and stage of OA at conventional radiography were collected at baseline and at the last follow-up. The Kellgren–Lawrence grading score [74] was used to assess the stage of OA. Data on the rate of failure and progression to TKA (total knee arthroplasty) were collected. Failures were defined as the recurrence of symptomatic meniscal tears confirmed by imaging, or the need for subsequential surgery.

### Assessment of the risk of bias

Two reviewers (**;**) independently evaluated the risk of bias of the extracted studies. Disagreements were solved by a third author (**). The software Review Manager 5.3 (The Nordic Cochrane Collaboration, Copenhagen) was used. The following endpoints were evaluated: selection, detection, performance, attrition, reporting, and other bias.

### Synthesis methods

The statistical analyses were performed by the main author (**) following the recommendations of the Cochrane Handbook for Systematic Reviews of Interventions [34]. For descriptive statistics, the IBM SPSS software version 25 was used. The arithmetic mean and standard deviation were used. The student *t* test was performed to assess baseline comparability, with values of *P* > 0.1 considered satisfactory. For the meta-analyses, the software Review Manager 5.3 (The Nordic Cochrane Collaboration, Copenhagen) was used. For continuous data, the inverse variance method with mean difference (MD) effect measure was used. For binary data, the Mantel–Haenszel method with odd ratio (OR) effect measure was used. The CI was set at 95% in all the comparisons. Heterogeneity was assessed using $\chi$^2^ and Higgins-*I*^2^ tests. If $\chi$^2^ > 0.05, no statistically significant heterogeneity was found, and a fixed model effect was used. If $\chi$^2^ < 0.05 and Higgins-*I*^2^ > 60% high heterogeneity was found, and a random model effect was used for analysis. Values of *P* < 0.05 were considered statistically significant. To assess the risk of publication bias, the funnel plot of the most commonly reported outcome was performed. Egger’s linear regression was performed through the STATA MP Software version 16 (StataCorp, College Station, USA) to assess funnel plot asymmetry, with values of *P* < 0.05 indicating statistically significant asymmetry.

## Results

### Study selection

**Fig. 1:**
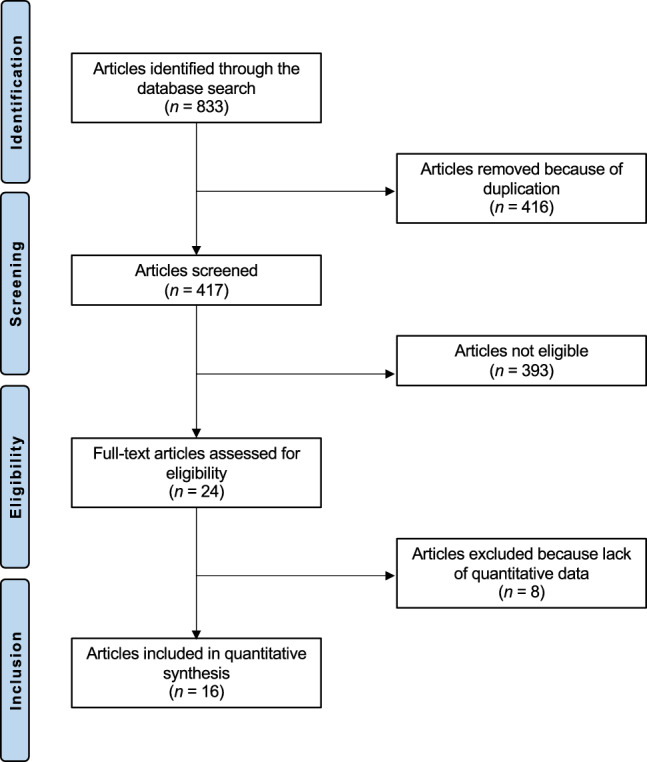
PRISMA flow chart of the literature search

The literature search resulted in 837 articles. Of them, 420 were excluded as they were duplicates. A further 389 articles were excluded as they did not match the eligibility criteria: study type and design (*N* = 285), not comparing meniscal repair versus resection (*N* = 97), augmentation with cell therapies (*N* = 10), and language limitations (*N* = 5). A further 8 studies were excluded as they missed quantitative data under the outcomes of interests. Finally, 20 comparative studies were included: 16 retrospective and four prospective clinical investigations. The results of the literature search are shown in Fig. 1.

### Analysis of publication bias

**Fig. 2:**
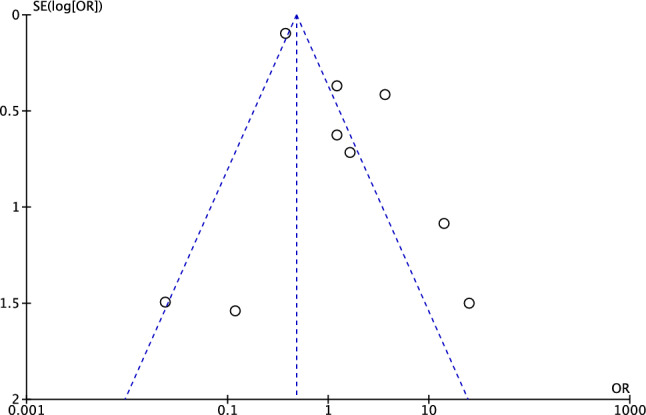
Funnel plot of the most reported outcome (failures)

To evaluate the risk of publication bias, the funnel plot of the most reported outcome (failure) was evaluated. The Egger’s test did not identify any statistically significant asymmetry (*P* = 0.9), indicating acceptable risk of publication bias. The funnel plot is shown in Fig. 2.

### Risk of bias assessment

**Fig. 3:**
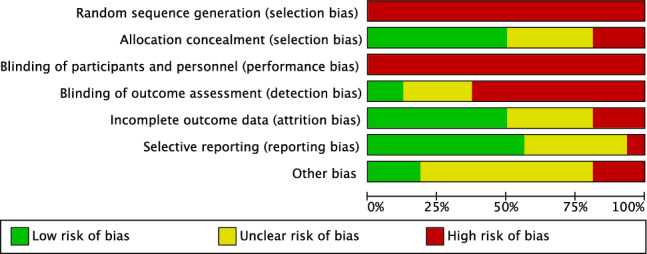
Cochrane risk of bias tool

Given the lack of randomised controlled trials included in the present investigation, the risk of selection bias was moderate to high. Few authors performed assessor blinding, leading to a moderate risk of detection bias. No authors performed patient blinding, which lead to a high risk of performance bias. The risk of attrition and reporting biases were moderate, as was the risk of other bias. Concluding, the risk of bias graph evidenced a moderate quality of the methodological assessment (Fig. 3).

### Study characteristics and results of individual studies

Data from 31,783 patients were collected. The mean BMI was 28.3 ± 3.2 kg/m^2^, and the mean age was 37.6 ± 14.0 years. The mean time elapsed from injury to surgery was 12.1 ± 10.2 months, and the mean medial joint width was 4.9 ± 0.8 mm. The generalities and demographics of the included studies are shown in Table 1.

**Table 1:** Generalities and patient baseline of the included studies (LoE: level of evidence)

Author, year	Journal	LoE	Follow-up (months)	Treatment	Patients (*n*)	Age (mean)	Women (%)
Bernard et al., 2020 [12]	*Am J Sports Med*	III	40.0	Repair	15	46.1	33.3
			58.0	Resection	15	48.8	33.3
Chung et al., 2015 [17]	*Arthroscopy*	III	72.0	Repair	37	55.5	89.2
			67.5	Resection	20	55.0	80.0
Deuthman et al., 2021 [23]	*Orthop J Sports Med*	III	100.8	Repair	79	17.7	29.1
				Resection	138	17.3	26.1
Dzidzishvili et al., 2022 [24]	*Orthop J*	III	27.0	Repair	30	52.2	
				Resection	35	56.0	
Hoshino et al., 2022 [36]	*J Orthop Sci*	II	24.0	Repair	139	25	55.4
				Resection	30	29	43.3
Kim et al., 2011 [46]	*Arthroscopy*	III	48.5	Repair	30	55.2	83.3
			46.0	Resection	28	57.4	85.7
Kim et al., 2019 [45]	*Arthroscopy*	III	39.2	Repair	21	55.9	12.5
			37.2	Resection	24	58.8	9.5
Lee et al., 2018 [49]	*J Orthop Sur*	III	19.4	Repair	70	42.2	35.7
			27.6	Resection	42	41.1	33.3
Lutz et al., 2015 [50]	*Orthop Traumatol Surg Res*	III	127.2	Repair	10	20.11	25
				Resection	22	38.9	
Persson et al., 2017 [71]	*Osteoarthritis Cartilage*	II	112.8	Repair	229	24.1	33.0
			133.2	Resection	2258	31.1	26.0
Phil et al., 2021 [75]	*Acta Orthop*	II	60.0	Repair	32	26.0	31.3
				Resection	118	32	34.0
Rockborn et al., 2000 [76]	*Knee Surg Sports Traumatol Arthrosc*	III	156.0	Repair open	30	26	20
				Resection	30	26	20
Shelbourne et al., 2003 [81]	*Am J Sports Med*	II	106.8	Repair	56	21.5	
			93.6	Resection	99	23.9	
Shelbourne et al., 2004 [82]	*Arthroscopy*	III	84.0	Repair	67	21.5	
			132.0	Resection	24	23.5	
Shrestha et al., 2022	*Kathmandu Univ Med J (KUMJ)*	III	12–30	Repair	43	34.3	42.0
				Resection	50	34.0	46.0
Sommerlath et al., 1992 [85]	*Int Orthop*	III	84.0	Repair open	25	27	28.0
				Resection	25		
Sochacki et al., 2020 [84]	*Am J Sports Med*	III	45.4	Repair	5516	29.9	40.7
			45.6	Resection	22,064	30.0	41.3
Stein et al., 2010 [87]	*Am J Sports Med*	III	106.0	Repair	42	31.3	38.1
				Resection	39	32.5	30.8
Su et al., 2022 [88]	*Cartilage*	III	41.4	Repair	21	62.1	81.0
			46.3	Resection	22	57.8	90.9
Ventura et al., 2023 [92]	*Cureus*	III	24.0	Repair	22	50.95	36.36
				Resection	22	53.41	54.55

### Baseline comparability

Between studies comparability at baseline was found in age, women, BMI, time from injury to surgery and length of follow-up, PROMs, medial joint width, and stage of OA. Baseline comparability is shown in Table 2.

**Table 2:** Comparison of the baseline demographic between the two groups

Endpoint	Repair (*N* = 6514)	Resection (*N* = 25,105)	*P*
Age	36.2 ± 14.6	38.7 ± 13.7	0.4
Women (%)	41.6 ± 22.6	42.0 ± 23.2	0.5
Follow-up (months)	69. 4 ± 40.1	73.5 ± 42.3	0.6
BMI (kg/m^2^)	27.3 ± 2.2	28.3 ± 2.6	0.5
Time from injury to surgery (month)	10.2 ± 8.8	14.1 ± 12.1	0.4
Lysholm score	56.1 ± 15.5	56.1 ± 15.7	0.5
IKDC	46.1 ± 15.0	57.2 ± 16.1	0.5
Tegner activity score	5.0 ± 2.2	5.1 ± 2.1	0.5
Medial joint width (mm)	5.1 ± 0.8	4.8 ± 0.9	0.3
Rate of OA grade I–II	74%	67%	0.3
Rate of OA grade III–IV	68%	53%	0.08

### PROMs

**Fig. 4:**
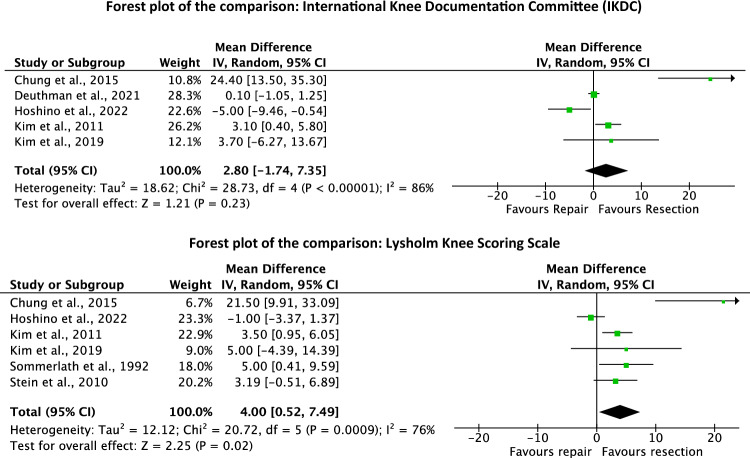
Forest plots of PROMs

The resection group demonstrated a greater Lysholm score (MD 4.0; 95% CI 0.52–7.49; *P* = 0.02). However, this difference does not exceed the MCID. No difference was found in IKDC (*P* = 0.2). These results are shown in Fig. 4.

### Failures

**Fig. 5:**
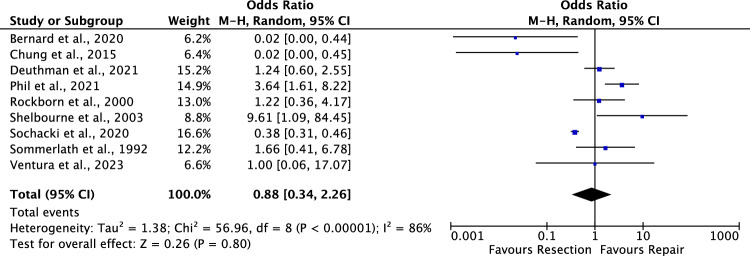
Forrest plots of the rate of failure

Nine studies reported data on failures at a mean of 63.00 ± 24.7 months [12, 17, 23, 75, 76, 81, 84, 85, 92]. No difference was found in the rate of failures (*P* = 0.8, Fig. 5).

### Progression to osteoarthritis

Six studies reported data on the rate of progression to total knee arthroplasty [12, 17, 24, 46, 84, 92] at a mean of 48.0 ± 14.7 months follow-up. The repair group evidenced a lower rate of progression to TKA (OR0.51; 95% CI 0.39–0.69; *P* = 0.0001).

**Fig. 6:**
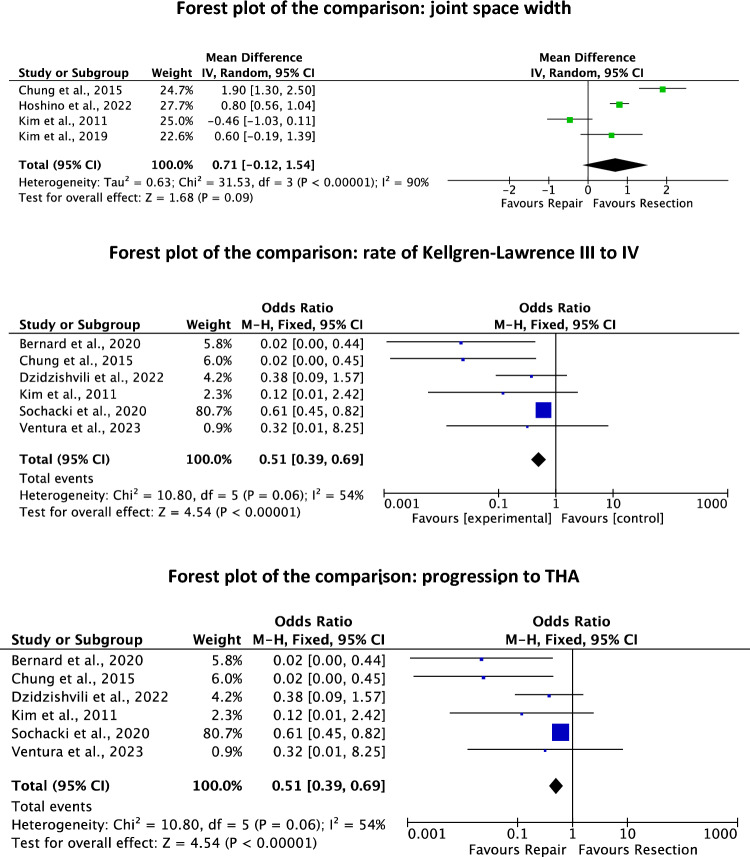
Forest plots of the rate of progression to osteoarthritis

Six studies reported data on the rate of advanced knee OA [12, 17, 24, 46, 84, 92] at a mean of 48.0 ± 14.7 months follow-up. The repair group evidenced a lower rate of advanced knee OA (OR 0.51; 95% CI 0.39–0.69; *P* = 0.0001). No difference was found in the mean joint space width (*P* = 0.09). These results are shown in Fig. 6.

## Discussion

According to the main findings of the present study, meniscal repair is associated with a lower progression to knee osteoarthritis at approximately six years of follow-up compared to partial meniscectomy. No difference in PROMs, medial joint width, and failures were evidenced.

Paxton et al. [70] reported in a systematic review that meniscal repair carries higher reoperation rate. No further between-groups differences were evidenced. Xu et al. [95], in a recent meta-analysis of seven studies, concluded that meniscal repair achieves greater PROMs and a lower failure rate. Faucett et al. [29] recently conducted a meta-analysis of nine studies evaluating acute tears of the medial meniscus root. Similarly, repair resulted in a lower rate of progression of OA compared to total resection or non-operative treatment [29]. Eseonu et al. [27], in a recent systematic review of 11 studies, evidenced that meniscal repair leads to a lower rate of progression of OA compared to meniscal resection and conservative management. The present meta-analysis improves the number of clinical investigations included for analysis and allows to analyse the rate of progression to OA in terms of joint space width, imaging, and rate of TKA.

Several factors influence the efficacy of meniscal repair. Geometry, location, alignment, site, size, and severity, exerts a major impact on the healing potential of the tear [26, 62]. However, the location of meniscal injury varies among the studies included in the present investigation. Eight studies exclusively investigated injuries of the medial meniscus [12, 17, 24, 45, 46, 81, 87, 88], and two, those of the lateral meniscus [23, 82]. Six of the studies that focus on medial injuries specify the location, including only injuries at the posterior root of the medial meniscus [12, 17, 24, 45, 46, 88]. Another eight studies included tears of both medial and lateral meniscus [36, 49, 50, 75, 76, 83, 85, 92]. Two studies did not specify the site of the injury [71, 84]. Injuries of the lateral and medial meniscus have different characteristics in terms of biomechanical function, aetiology, risk factors, and concomitant injuries [48, 72]. Given the lack of quantitative data on the outcomes of interest, it was not possible to analyse injury pattern separately.

The menisci are classically divided into three zones that differ in terms of vascularisation and metabolic activity [4, 5, 19]. Tears at the inner edge show the lowest repair capacity given the avascular nature of the tissue [18]. Initially, tears in the white and red–white zones were thought to be only partially suitable for repair procedure [38]. The described supply zones of the meniscus are considered in seven included studies of the present meta-analysis [36, 49, 71, 75, 76, 81, 87]. Stein et al. [87], Shelbourne et al. [81], and Hoshino et al. [36] reported tears in the red–red and red–white zones, whereas ruptures in the white–white zones underwent partial meniscectomy. The remaining studies do not describe in detail the tear location in relation to the vascular zones [12, 17, 23, 25, 45, 46, 82–85, 88, 92]. Although removal or partial resection of the meniscus is preferred for tears located in the less vascularised zones, there are increasing reports of successful repairs in the critical zones as well [18, 77]. These results indicate that repair should be attempted in all three zones if at all possible [18, 65].

Depending on the location and type of meniscal tear, inside-out, outside-in, and all-inside techniques are used [68]. The inside-out technique is still considered the gold standard for a variety of tear types [33]. The outside-in technique has become less popular compared to the inside-out technique [63, 93]. Both techniques involve a mini-incision and suturing the meniscus to the capsule [73, 86]. The all-inside technique offers many options, including arthroscopic suture tying and the availability of numerous absorbable fixation devices such as arrows, fastens, darts, and staples [20, 52, 53]. In particular, the ease and associated shorter operating times have led to the great popularity of the all-inside technique [30, 31, 91]. The influence of different repair techniques on clinical outcomes is not conclusive. Stein et al. [87] and Shelbourne et al. [81, 82] used the standard inside-out method. In four studies [49, 50, 83, 88], the outside-in or all-inside technique was used, depending on the location of the tear. Three studies considered all common repair techniques [23, 75, 92]. All studies dealing exclusively with the repair of root tears used the transtibial pull-out method, a modified inside-out technique [12, 17, 24, 45, 46]. No detailed information on the procedure used was found in two of the included studies [36, 84]. Two studies performed open meniscal repair [76, 85]. Open meniscus repair is indicated in selected patients with complex posterior horn tears in combination with an extremely narrow medial compartment to facilitate access [42, 78].

The majority of included studies excluded patients with concurrent knee ligament injury [12, 17, 23, 24, 45, 46, 50, 75, 76, 81, 82, 84, 85, 87]. Person et al. [71] did not exclude ACL injuries, but performed statistical adjustments, when necessary, for age and sex, before evaluating the results. However, meniscal injuries are frequently associated with cruciate ligament injuries, especially tears of the anterior cruciate ligament [55]. When ACL reconstruction is performed concomitantly with the repair of meniscal tears, significantly better healing of meniscal tears is reported [16]. Conversely, meniscal repair leads to increased ACL stability, and ACL reconstruction also benefits from meniscal repair [43]. Therefore, seven of the included studies required ACL injury as an inclusion criterion [36, 49, 81–83, 88, 92]. The applicability of the results to knees without ACL damage is uncertain.

Between studies heterogeneity in the resection technique are evident. Only three studies [23, 83, 84] did not define the type of meniscectomy in detail. The other authors [12, 17, 24, 36, 45, 46, 49, 50, 71, 75, 76, 81, 82, 85, 87, 88, 92] conducted a partial meniscectomy [1, 7, 94]. An arthroscopic procedure for partial meniscectomy is used in five studies [50, 71, 75, 76, 87]. Despite the results of the repair and controversies in the current literature, partial resection continues to be indicated in complex, degenerative, avascular tears, or following failure of a previous meniscal repair [52, 59]. Partial meniscectomy is believed to achieve faster recovery and symptoms remission, is simple to perform and requires a short operation time [8, 13, 49].

Additional limitations are evident. Patient age significantly influences the aetiology of meniscus tears, but it is also an important factor regarding the development of OA. Most studies included patients aged 20–30 years [36, 71, 75, 76, 81, 82, 84, 85, 87]. Data from patients aged 40 to 60 years or older are considered in eight studies [12, 17, 24, 45, 46, 49, 88, 92]. Lutz et al. [50] and Shrestha et al. [83] included patients aged 20–40 years. On the other hand, Duethman et al. [23] included patients aged 17 years old. This different age may lead to selection bias. Further, heterogeneity in the outcomes of interest was evident. This can be attributed to the fact that there are no standard criteria to define successful tear healing. Most authors used radiographs for the diagnosis and follow-up [17, 24, 36, 45, 46, 50, 76, 81, 82, 85]. Magnetic resonance imaging was used in four studies [24, 45, 46, 88]. Second-look arthroscopy was performed in two studies [45, 46]. Five studies exclusively referred to PROMs to assess outcomes [71, 75, 83, 84, 92]. Most authors did not specify whether-radiographs of knees were undertaken under weight-bearing condition. Most of the current studies are retrospective [17, 24, 45, 49, 50, 81–83, 88, 92]. Level I studies are completely missing, only four have an evidence level of II [36, 71, 75, 81], and 80% (16 of 20) of the included studies achieve level III evidence [12, 17, 23, 24, 45, 46, 49, 50, 76, 82–85, 87, 88, 92]. Further long-term high-quality studies considering the limitations mentioned above are needed to confirm the advantage of meniscal repair over resection. Moreover, the importance of adequate post-surgery rehabilitation programmes should also be investigated in long-term high-quality larger-scale studies.

## Conclusion

Meniscal repair is associated with a lower progression to knee osteoarthritis at approximately six years of follow-up compared to partial meniscectomy. No difference in PROMs, medial joint width, and failures were evidenced.

## Data Availability

The datasets generated during and/or analysed during the current study are available throughout the manuscript.
